# Piscine Orthoreovirus 3 Is Not the Causative Pathogen of Proliferative Darkening Syndrome (PDS) of Brown Trout (*Salmo trutta fario*)

**DOI:** 10.3390/v11020112

**Published:** 2019-01-28

**Authors:** Robert Fux, Daniela Arndt, Martin C. Langenmayer, Julia Schwaiger, Hermann Ferling, Nicole Fischer, Daniela Indenbirken, Adam Grundhoff, Lars Dölken, Mikolaj Adamek, Dieter Steinhagen, Gerd Sutter

**Affiliations:** 1 Institute for Infectious Diseases and Zoonoses, Department for Veterinary Sciences, LMU Munich, 80539 Munich, Germany; daniela.arndt@micro.vetmed.uni-muenchen.de (D.A.); martin.langenmayer@lmu.de (M.C.L.); gerd.sutter@lmu.de (G.S.); 2 Bavarian Environment Agency, Unit Aquatic Toxicology, Pathology, 82407 Wielenbach, Germany; julia.schwaiger@lfu.bayern.de (J.S.); hermann.ferling@lfu.bayern.de (H.F.); 3 Institute for Medical Microbiology, Virology and Hygiene, University Medical Center Hamburg Eppendorf, 20246 Hamburg, Germany; nfischer@uke.de; 4 Heinrich Pette Institute, Leibniz Institute for Experimental Virology, 20251 Hamburg, Germany; daniela.indenbirken@leibniz-hpi.de (D.I.); adam.grundhoff@leibniz-hpi.de (A.G.); 5 Institut für Virologie und Immunbiologie, Julius-Maximilians-Universität Würzburg, 97078 Würzburg, Germany; lars.doelken@uni-wuerzburg.de; 6 Fish Disease Research Unit, Institute for Parasitology, University of Veterinary Medicine, 30559 Hannover, Germany; mikolaj.adamek@tiho-hannover.de (M.A.); dieter.steinhagen@tiho-hannover.de (D.S.)

**Keywords:** proliferative darkening syndrome, black trout syndrome, piscine orthoreovirus, orthoreovirus, brown trout, *Salmo trutta fario*, next generation sequencing, RT-qPCR

## Abstract

The proliferative darkening syndrome (PDS) is a lethal disease of brown trout (*Salmo trutta fario*) which occurs in several alpine Bavarian limestone rivers. Because mortality can reach 100%, PDS is a serious threat for affected fish populations. Recently, Kuehn and colleagues reported that a high throughput RNA sequencing approach identified a piscine orthoreovirus (PRV) as a causative agent of PDS. We investigated samples from PDS-affected fish obtained from two exposure experiments performed at the river Iller in 2008 and 2009. Using a RT-qPCR and a well-established next-generation RNA sequencing pipeline for pathogen detection, PRV-specific RNA was not detectable in PDS fish from 2009. In contrast, PRV RNA was readily detectable in several organs from diseased fish in 2008. However, similar virus loads were detectable in the control fish which were not exposed to Iller water and did not show any signs of the disease. Therefore, we conclude that PRV is not the causative agent of PDS of brown trout in the rhithral region of alpine Bavarian limestone rivers. The abovementioned study by Kuehn used only samples from the exposure experiment from 2008 and detected a subclinical PRV bystander infection. Work is ongoing to identify the causative agent of PDS.

## 1. Introduction

The brown trout (*Salmo trutta fario*) is a predatory fish of the family *Salmonidae*. Its range of distribution covers nearly the entirety of Europe, and its natural habitats are fast flowing, cool, oxygen-rich waters. Since the 1980s, an annually recurring die-off of brown trout takes place in the rhithral region of alpine Bavarian limestone rivers. In the affected river sections, the mortality rates reach almost 100%. Typically, diseased brown trout develop a dark or almost black color before they die. Therefore, the disease was named “proliferative darkening syndrome” (PDS) or “black trout phenomenon”. Similar disease events were observed in Austria and Switzerland. However, black pigmentation in fish is a rather unspecific symptom, reflecting a response to various factors, and is not pathognomonic for a particular etiology [1,2]. Therefore, it remains unclear whether the term “PDS” describes the same type of disease in different pre-alpine regions.

**Figure 1:**
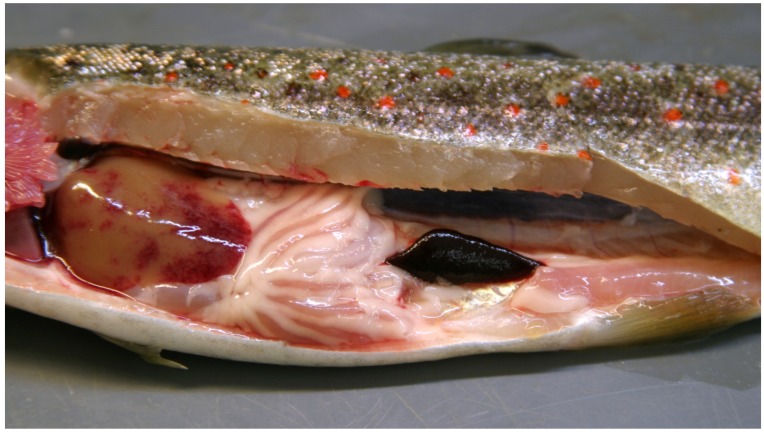
A brown trout with “proliferative darkening syndrome”. Note the hepatic hemorrhages and the enlarged congested spleen. (Photograph provided by the Bavarian Environment Agency).

The PDS observed in the Bavarian rivers shows some interesting characteristics. The clinical disease and subsequent death of the brown trout is exclusively observed in late summer (August to September). However, the syndrome only appears if the respective fish were exposed to water of the affected river sections during May and June. In the final stage of PDS, typical clinical signs include besides the darkening of the skin, apathy and highly increased respiratory rates. The pathological lesions comprise pronounced anemia and leucopenia, hepatic hemorrhages, splenic enlargement, and ascites (Figure 1). Histological features include necrohemorrhagic hepatitis and lymphocytic depletion of the spleen [3].

Recently, Kuehn and colleagues [4] reported that a piscine orthoreovirus (PRV) is the causative agent of the PDS of brown trout using a high throughput RNA sequencing (RNA-seq) approach. In that study, brown trout specimens were exposed in the Iller, a river which is regularly affected by PDS. Afterwards, samples were collected at various time points until the occurrence of clinical signs of PDS. Using transcriptomic analysis and RT-qPCR, the strong induction of immune-associated genes was observed supporting an infectious nature of the disease. Samples from fish with the corresponding expression profile were used for next-generation sequencing (NGS), which resulted in the detection of PRV RNA.

PRV is a non-enveloped virus with a segmented (L1-3, M1-3, S1-4), double stranded RNA genome, which belongs to the genus *Orthoreovirus*, subfamily *Spinareovirinae* within the family *Reoviridae*. At least three subtypes of the virus exist: PRV-1 is frequently found in farmed Atlantic salmon (*Salmo salar*) and is associated with the heart and skeletal muscle inflammation (HSMI) syndrome but also can be detected in apparently healthy fish as well as in other species of the genera *Salmo* and *Oncorhynchus* including sea trout (*Salmo trutta*) [5,6,7,8]. The pathogenesis of HSMI is only partially understood. Interestingly, erythrocytes are important target cells for PRV-1, but infection does not result in severe anemia [9]. In cohabitant infection trials, the virus was detectable in blood samples after 4–6 weeks [9] and clinical symptoms occurred after 8–10 weeks [10]. After disease outbreaks, subclinical persistence of the virus in the fish population has been described by several studies [7,8,11]. In Japan, Takano and colleagues described PRV-2 as causative agent of the erythrocytic inclusion body syndrome (EIBS) of Coho salmon (*Oncorynchus kisutch*). In contrast to HSMI and PRV-1, in EIBS-affected fish, the level of anemia is corresponding with the level of viral replication in the blood [12]. Recently, Dhamatharan and colleagues characterized the molecular and antigenic properties of PRV-3, which the occurrence of was already demonstrated in Norway, Chile, Denmark, Italy, Scotland, and Germany [13,14,15,16]. PRV-3 was described to cause HSMI-like disease in rainbow trout (*Onchorhynchus mykiss*) [14,15]. Besides typical lesions in the heart and skeletal musculature, the vacuolization and necrosis of hepatocytes, swollen kidneys and spleens, anemia, and ascites were observed [14]. However, consecutive infection experiments failed to reproduce these findings [17]. Recently, the occurrence of PRV-3 in Germany has been demonstrated. The virus was detected in farmed rainbow trout showing darkening of the skin, lethargic behavior, hemorrhages in the muscles and the heart, and increased mortality. However, in this case, infection with *Aeromonas salmonicida* subsp. *salmonicida* and not with PRV-3 was suggested as the main cause of the clinical signs [16].

Because of the high sequence identity to the reference genome (GenBank no. MG253807-MG253816), the PRV described by Kuehn and colleagues has to be classified as PRV-3. While PRV-3 was indeed a candidate for PDS, we show here that PRV-3 is not the causative agent of PDS in pre-alpine rivers of Bavaria. In contrast, it represented a bystander infection that was also observed in the control fish of the respective experiment but was not observed in the diseased fish from a follow-up cohort in the year 2009.

## 2. Materials and Methods

### 2.1. Sample Origin

**Figure 2:**
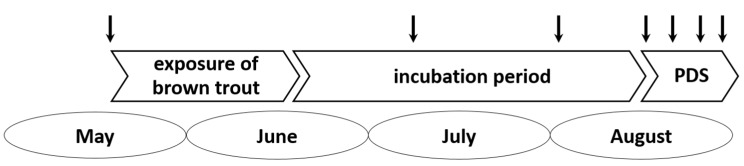
The timeline of the PDS exposure experiment in 2009. Brown trout were exposed to the water of the PDS affected river Iller during May and June. The first signs of PDS appeared in mid-August. The arrows indicate the time points of sampling.

All animal experiments were approved by the competent authority (Government of Upper Bavaria, AZ 55.2-1-54-2531.2-19-02) and were carried out according to the requirements of the German animal welfare legislation. In order to identify the causative agent of pre-alpine PDS, a well-established study design was used. In the years 2008 and 2009, 1.5-year-old brown trout were exposed to the water of PDS-affected sections of the river Iller in a bypass-system during the months of May and June. After the exposure period, the fish were transferred to the quarantine station of the Bavarian Environment Agency. In aquariums supplied with spring water, they were continuously observed until the appearance of clinical signs of PDS (August–September). Diseased fish were anaesthetized in MS222 (Tricaine, Pharmaq Ltd. 10 g/ 100 L) and subsequently killed by decapitation. A mock group was kept in spring water all the time. From the experiment in 2009, we were able to investigate samples from ten control fish (taken in May), 19 fish exposed to Iller water but showing no signs of PDS yet (taken in the incubation period in July and early August), and 20 fish demonstrating typical signs of PDS (mid-/end of August) (Figure 2). From the experiment in 2008, from which Kuehn and colleagues [4] obtained the samples for their investigations, the organs of eight fish with clinical signs of PDS and of four control fish were available. For the exposure experiment in 2008, the brown trout from the breeding unit of a local fish farm were used. In 2009, the fish for the experiment were derived from the hatchery of the Bavarian Environment Agency in Wielenbach.

### 2.2. Pathohistological Investigation

Spleen and liver samples of the control and PDS-affected fish were routinely fixed, processed, embedded, cut, stained with haemalum-eosin, and subsequently examined by light microscopy.

### 2.3. Next-Generation Sequencing

For the next-generation sequencing, we used liver and kidney samples from 49 brown trout from the exposure experiment in 2009. Ten control fish were not exposed to Iller water, 20 animals were sampled during the incubation period, and the remaining brown trout were investigated during the clinical PDS period. Tissue samples were homogenized in PBS using Precellys® 2 mL soft tissue homogenizing ceramic beads and a Precellys 24 homogenizer (Peqlab, Erlangen, Germany) using the following conditions: 2 × 10 s at 5000 rpm. The homogenized tissue was briefly centrifuged, and the supernatant was subsequently used for RNA extraction using the QIAamp RNeasy Mini Kit (Qiagen, Hilden, Germany). RNA was eluted in 80 µL RNAse free water, and DNAse digest was performed using the DNAfree Kit (Life technologies, Carlsbad, CA, USA). The RNA of four or five organ samples was pooled, and quality of the RNA pools was monitored using Agilent 2100 Bioanalyzer and RNA 6000 Nano RNA chips. The rRNA was depleted using the Ribo-Zero Gold rRNA Removal Kit (Human/Mouse/Rat) (Illumina, San Diego, CA, USA) following the manufacturer’s instructions. RNA Illumina NGS libraries were prepared from each RNA pool (pool 1–20) using a modified protocol of the SCRIPT SEQ^TM^ v2 RNA Seq Kit (Illumina, San Diego, CA, USA) which was described recently [18]. All libraries were multiplexed sequenced on an Illumina NovaSeq 6000 system (2 × 150 cycles on a paired-end protocol; S4 flowcell) according to the manufacturer’s protocol. Each RNA pool was sequenced at a depth of approximately 128 million paired reads (2.57 × 10^9^ total reads across all pools).

The bioinformatic analysis of sequencing reads was performed using the pathogen detection pipeline described before [18]. Briefly, the host reads (between 93.13% to 97.15% of all reads per pool, average 96.16%) were subtracted by aligning the reads to the latest genome assembly of *Salmo salar* (Atlantic salmon, GCF_000233375.1) using Bowtie2 (v2.3.1). Trinity (r2013-02-25) was used to assemble contigs from the reads not producing significant host alignments. The contigs assembled were subsequently filtered for sequences of a minimal length of 400 bp. For taxonomic classification, the filtered contigs were aligned to the NCBI nt database using the blast+ package (v2.6.0). To investigate the potential presence of PRV-3 reads that may had failed to assemble into larger contigs, we also directly mapped the original reads to the 17 genomic sequences (GenBank numbers MH513858 to MH513874) reported by Kuehn and colleagues [4] using Bowtie2 (v2.3.1).

### 2.4. Detection and Sequencing of PRV3 RNA

RNA was isolated from the organ samples using the QIAamp RNeasy Mini Kit (Qiagen, Hilden, Germany) according to the manufacturer’s instruction. To denature viral dsRNA, the eluate was incubated for 10 min at 95 °C before RT-PCR. For all RT-PCRs, the QuantiTect probe RT-PCR kit (Qiagen, Hilden, Germany) was used. We designed a TaqMan qPCR assay amplifying a target sequence of the S1 segment for the detection of PRV-3. The following oligonucleotide primers and concentrations were used: primer PRV3 S1 F_112_: ATC TCT GGC ACC ACA AGA TTT (800 nM), primer PRV3 S1 R_192_: GAC CAT AGC AGG CTT AGC RTT A (800 nM), and PRV3 S1 probe_167_: FAM-AGA CAG ACC AAY CCK ATG CCC GC-BHQ1 (300 nM). The thermal profile of the PCR was 50 °C for 30 min; 95 °C for 15 min; and 42 cycles of 95 °C for 15 s, 57 °C for 20 s, and 68 °C for 40 s. Additionally, we amplified a 424 bp sequence of the S1 segment using a pair of primers (S1-1 F: TAG GAC GGC GAC AAC TAC TG, S1-1 R: TCT AAG GCG TCG CTT AGC TT) published by Kuehn and colleagues [4]. The PCR products were controlled by agarose gel electrophoresis and sequenced using the PCR primers and the sequencing service of Eurofins Genomics (Ebersberg, Germany). For the alignment and analysis of the obtained sequences, the DNASTAR Lasergene software was used.

## 3. Results

### 3.1. Clinical Signs and Pathologic Lesions

**Figure 3:**
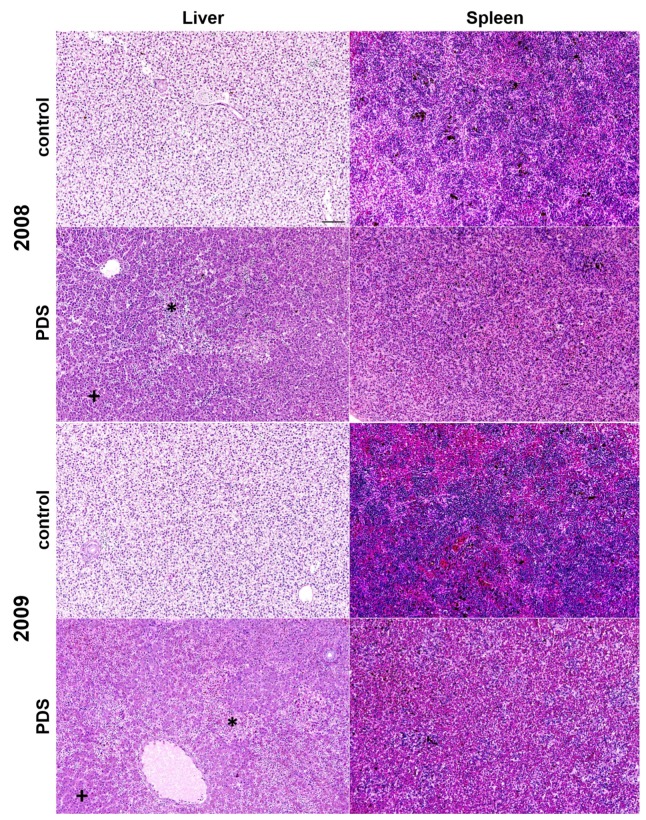
Organ lesions of brown trout of the exposure experiments in 2008 and 2009 with proliferative darkening syndrome (PDS) compared to the control animals. The livers of PDS-affected brown trout displayed multifocal random bridging liquefactive necrosis (*) and viable hepatocytes with eosinophilic cytoplasm (diminished glycogen storage, +). The livers of the control animals displayed physiologic diffuse clear cytoplasmic vacuolation of hepatocytes (glycogen storage) without necrotic foci; the bar = 100 μm (same magnification in all pictures). The spleens of PDS-affected brown trout displayed a pronounced depletion of lymphocytes when compared to the spleens of the control animals.

The clinical signs of brown trout affected by PDS included apathy, decreased movement, separation from the group, anorexia, and darkening of the skin in both years. The livers and spleens of brown trout affected by PDS had similar lesions in both exposure experiments (2008 and 2009). Macroscopic and histologic lesions in livers and spleens of brown trout affected by PDS were indistinguishable between both years. In the livers, multifocal to coalescing random areas of liquefactive hepatocyte necrosis were accompanied by hemorrhages (necrohemorrhagic hepatitis; about 10–15% of the tissue was affected). The remaining viable hepatocytes displayed diminished glycogen stores. Spleen histology was characterized by multifocal to diffuse the depletion of lymphocytes from white pulp areas (Figure 3). In both organs, there were multiple foci of erythrophagocytosis. In contrast, control fish not exposed to river water did not show either typical clinical signs of PDS nor macroscopic or histologic organ lesions in both exposure experiments.

### 3.2. Next-Generation Sequencing

We constructed strand-specific libraries from the RNA pools generated from the RNA isolated from the liver and kidney tissue of the control (four pools) and diseased animals (16 pools). These RNA libraries were multiplex-sequenced at an average depth of 128 million paired reads per pool, producing a total of approximately 2.6 billion reads. The primary sequence reads were aligned to the *Salmo salar* reference genome to subtract the reads of host origin. The filtered reads were subjected to de novo assembly and taxonomic binning with a previously validated pathogen detection pipeline [18]. None of the approximately one million assembled contigs (145,842 and 862,117 in the control and PDS pools, respectively) were classified as piscine orthoreovirus or any other member of the reoviridae family. Given that only 289 of the 337 million sequencing reads in the report by Kuehn and colleagues had mapped to PRV-3 [4], we also considered the possibility that low read abundance of specific reads in our dataset may have hindered the assembly of reoviral contigs or that the length of the resulting contigs may have been below the cutoff value of our analysis pipeline (400 nt). Therefore, we additionally mapped our reads directly to the PRV-3 sequences reported by Kuehn and colleagues. Again, we were unable to detect reads mapping to any of the segments. Hence, although the total base space (3.85 × 10^11^ nt) covered by our sequencing analysis was more than 10 times larger than that of Kuehn and colleagues, we were not able to detect PRV-associated sequences in any of the samples from the exposure experiment in 2009.

### 3.3. PRV3 Detection Using qPCR

In accordance with Kuehn and colleagues [4], we tested the liver samples of brown trout for PRV-3 by RT-qPCR. From the 2009 sample collection, we investigated ten control fish, 20 animals sampled during the incubation period, and 17 brown trout showing clinical and pathological signs of PDS. In agreement with our NGS data, PRV-3 RNA was not detectable in any of the specimen. We, thus, also investigated the liver samples from the fish of the 2008 study analyzed by Kuehn and colleagues [4]. Here, PRV-3 was present in all the PDS fish. Additionally, we tested the kidney and spleen samples from these animals, which also showed moderate levels of PRV-3 RNA. However, when we tested the liver, kidney, and spleen samples from the control trout not exposed to the water of the Iller from the same experiment, similar viral loads were detectable in all four fish tested (Table 1).

To verify the positive results of the RT-qPCR, we amplified and sequenced a 424 bp fragment of the PRV-3 S1 open reading frame of six PDS brown trout and three mock control fish. All nine nucleotide sequences showed a 100% identity with the reference sequence (GenBank no. MH513870) published by Kuehn and colleagues [4]. We conclude that PRV-3 is not responsible for the alpine proliferative darkening syndrome but represented a bystander infection of the 2008 experiment.

## 4. Discussion

The “proliferative darkening syndrome” is a remarkably species-specific disease of the brown trout, which is annually occurring in various alpine Bavarian limestone rivers. Despite decades of investigations and efforts to identify the cause of this phenomenon, the etiology and the causative agent are still unknown. Nevertheless, the seasonal nature and clinical picture implies that PDS is caused by an infectious agent. Next-generation RNA sequencing (RNA-seq) is a very powerful tool for the discovery of novel or unknown pathogens [19,20,21,22,23].

Kuehn and colleagues [4] employed RNA-seq and described a detection pipeline for unknown pathogens, which was used to investigate the samples from a PDS exposure experiment realized in 2008 at the river Iller. They identified PRV-3 as the causative agent of PDS. Similar to Kuehn and colleagues, we used RNA-seq of a sample collection obtained from a comparable exposure experiment in 2009. We analyzed a total of 2.57 billion read fragments and assembled more than one million contigs with a length of 400 bp or more. We performed an extensive analysis of the assembled contigs using a validated pipeline which performs Blastn, Blastp, and HMMER searches to identify pathogen signatures and additionally directly mapped the primary reads to PRV-3. However, we could not detect any PRV-specific sequences. Moreover, a highly sensitive and PRV-3-specific RT-qPCR failed to detect the virus in the affected brown trout demonstrating the typical clinical signs and pathologic lesions of PDS. We conclude that PRV was not the causative agent inducing PDS in brown trout in the river Iller in 2009.

On the other hand, RT-qPCR detected PRV-3 RNA (mean Cq values of 29, 28, and 26 for liver, kidney, and spleen, respectively) in all available PDS-affected fish from the experiment in 2008. However, similar amounts of the virus were also detectable in the uninfected control fish, which were not exposed to the water of the PDS-affected river sections of the Iller and which did not show any clinical or pathological sign of PDS. In 2008, the brown trout used in the exposure experiment were obtained from a local hatchery which housed the fish in surface water. In contrast, the experimental animals from 2009 were raised in spring water in a breeding unit of the Bavarian Environment Agency in Wielenbach. We conclude, that all fish of the 2008 experiment were infected with PRV-3, which thus represented a subclinical bystander infection, likely having occurred already in the hatchery before the exposure experiment. This is in accordance with previous reports of PRV-1 subclinical infections with moderate viral loads [11].

For PRV-1 and -2 it has been demonstrated, that the virus infections can be transmitted experimentally or from fish to fish [9,10,12]. Moreover, recently, Hauge and colleagues [17] reported the effective transmission of PRV-3 to naïve cohabitants in an infection study with rainbow trout. In contrast to these findings, the transmission of PDS has failed in cohabitant trials [3]. The proper fulfilling of Koch’s postulates, to prove the causative relation between an infectious agent and a disease, is extremely important but sometimes very difficult. This is particularly true in cases of multifactorial diseases or for pathogens with a complex life circle, for example those using intermediate host(s). Kuehn and colleagues argued that immune response gene expression profiles, which were analyzed in their study, indicate that PRV-3 is the likely causative of PDS. Indeed, an analysis of the transcriptional biomarkers might improve our knowledge of the disease pathogenesis or might become relevant for diagnostic procedures in future [24]. At present, however, this kind of analysis can at best suggest an infectious or inflammatory disease in general. It must not be considered as a replacement for the requirements to investigate a pathogen spread within an affected host, to demonstrate an association of the pathogen with the observed tissue lesions, and most importantly, to show the presence and transmission of the disease in infection experiments with relevant control groups.

In summary, we demonstrated that PRV-3 is not the causative agent of the proliferative darkening syndrome of brown trout in the rhithral region of alpine Bavarian limestone rivers. Work is ongoing to identify the causative agent of PDS based on the two sets of RNA-seq experiments.

## Figures and Tables

**Table 1:** PRV-3 in the brown trout from the exposure experiment in 2008. The virus specific RNA in the liver, kidney, and spleen was detected using RT-qPCR (Cq values are shown).

	Brown Trout with PDS									Mock Control			
	A	B	C	D	E	F	G	H	I	1	2	3	4
liver	29	29	27	29	30	30	30	30	30	30	30	28	31
kidney	24	26	25	30	28	27	30	28	29	31	33	27	27
spleen	23	23	24	27	27	27	27	28	27	29	26	25	26
